# Use of shear wave velocity for assessing engineering properties of compacted bentonite after swelling

**DOI:** 10.1038/s41598-023-42779-7

**Published:** 2023-09-21

**Authors:** Mintae Kim, Changho Lee, Jang-Un Kim, Hyunwook Choo

**Affiliations:** 1 School of Civil, Environmental and Architectural Engineering, Korea University, Seoul, 02841 South Korea; 2 Department of Civil Engineering, Chonnam National University, Gwangju, 61186 South Korea; 3 Department of Civil and Environmental Engineering, Hanyang University, Seoul, 04763 South Korea

**Keywords:** Civil engineering, Energy infrastructure

## Abstract

The characteristics of compacted bentonite after swelling determine the long-term stability of barrier systems. Due to the fact that the current stress level is the most important variable in determining the performance of engineered geosystems, this study aims to investigate the stress states and the consequent change in engineering properties of compacted bentonites after swelling. A series of vertical and horizontal swelling pressure tests were performed for compacted bentonites with varying initial dry unit weights at varying pore fluid concentrations. The compacted bentonite samples after swelling were loaded to investigate the changes in lateral stress and deformability. In addition, the shear wave velocity was continuously measured during and after swelling processes. The results of this study demonstrate that the swelling pressure increased with increasing dry unit weight of tested materials and decreasing pore fluid concentrations. The changes in lateral stress and void ratio of compacted bentonite after swelling were only measurable when the applied vertical stress was greater than the swelling pressure, reflecting that the swelling pressure cancels out the externally applied stress. Most notably, this study reveals that the initiation and termination of the swelling process and the change in engineering properties of compacted bentonite after swelling can be determined by measuring shear wave velocity.

## Introduction

Compacted bentonites have been considered as engineering materials for barriers in the deep geological repositories of radioactive waste or for landfill liners because of their characteristics of high swelling potential and swelling pressure^1^. Owing to the complexity of the behavior of compacted bentonite, its swelling characteristics have been extensively studied over several decades to elucidate the effects of mineralogy and microstructure of bentonite, initial water content, dry unit weight, type and concentration of infiltration solution, and temperature on swelling pressure or swelling strain^2–18^.

Previous studies have been conducted on the swelling behavior of compacted bentonite while the compacted bentonite expands; however, studies on the behavior of compacted bentonite after swelling are very limited. As the deep geological repositories for high-level nuclear waste or municipal solid waste landfill liners are designed and constructed for long-term operation, studies on the characteristics of the compacted bentonite after swelling must be conducted to ensure the stability of barrier systems. Because the performance of engineered geosystems is primarily determined by the current stress level, the determination of the lateral stress or coefficient of lateral stress at rest (*K*_*0*_) is critical^19^. However, the determination of in-situ stress levels from traditional geotechnical experiments is challenging because of the difficulty in accessing deep geological repositories or landfill liners. Therefore, non-destructive testing methods would be ideal for investigating the characteristics of compacted bentonite after swelling.

The measurement of the shear wave velocity (*V*_*s*_) provides informative geophysical characteristics of soils; therefore, many studies have been conducted to explore the application of *V*_*s*_ to the assessment of engineering properties of soils^20–26^. In particular, *V*_*s*_ is determined by the mean effective stress, leading to the measurement of *V*_*s*_ giving an indication of stress levels of in-situ soils. Furthermore, the *V*_*s*_ is dependent on the interaction between soil particles, leading to the fact that the swelling process may be monitored by the continuous measurement of *V*_*s*_.

Therefore, in this study, the characteristics of compacted bentonite were experimentally investigated during and after swelling using *V*_*s*_. The compacted bentonite specimens with varying dry unit weights were prepared under varying NaCl pore fluid concentrations. Vertical and horizontal swelling pressure tests were performed separately during swelling, and *V*_*s*_ was continuously measured. After the completion of swelling, the sample was loaded to examine the variations of the deformation, lateral stress, and *V*_*s*_ of the compacted bentonite after swelling. Considering the complexity of the characteristics of the compacted bentonite, these test results can provide valuable insights into understanding the characteristics of compacted bentonite during and after swelling using the measurement of *V*_*s*_.

## Experimental program

Vertical and horizontal swelling pressure tests at constant volume were performed for compacted bentonite with varying NaCl pore fluid concentrations and dry unit weights. Bender element tests were also executed for the measurement of shear wave velocity to investigate the characteristics of compacted bentonite during and after swelling. Table 1 indicates the experimental program performed in this study.

**Table 1:** Experimental program.

Type	Dry unit weight (kN/m^3^)	NaCl concentration (mol/L)			
		0 M	0.1 M	0.5 M	1.0 M
Vertical swelling test	11.77	- Measure the vertical swelling pressure of compacted bentonite during swelling at constant volume			
	12.75				
	13.73				
Thin wall oedometer test	11.77	- Measure the horizontal swelling pressure and shear wave velocity of compacted bentonite during swelling at constant volume - Measure the horizontal stress change and shear wave velcotiy of compacted bentonite after swelling			
	12.75				
	13.73				

### Test soil and sample preparation

The test soil used in this study is commercially available high plastic clay (USCS group symbol: CH) with a specific gravity (*G*_*s*_) of 2.51. The liquid limit (LL) and plastic limit (PL) were 89.25% and 38.12%, respectively. Table 2 summarizes the index properties of the tested soil. Based on semi-quantitative analysis (Bruker-Diffrac.EVA) of XRD results, the main mineral type was determined to be montmorillonite, and other mineral types are also reported in Table 2. The ratio between exchangeable Na^+^ and Ca^2+^ was 0.34, determined by ICP-OES (Varian 720-ES), indicating that the bentonite employed in this study was Ca-bentonite. Ca-bentonite is a representative bentonite distributed in Korea; thus the selection of Ca-bentonite reflected the regional characteristics.

**Table 2:** Index properties of test soil.

Soil type	Calcium bentonite
Specific gravity (G_s_)	2.51
Liquid limit, LL (%)	89.24
Plastic limit, PL (%)	38.12
Specific surface area (m^2^/g)*	260.56
Cation exchange capacity, CEC (meq/100 g)	90.1
Exchangeable cations (meq/100 g)	Na = 19
	Ca = 56.4
	Mg = 10.2
	K = 4.5
Maximum dry density, MDD (kN/m^3^)	11.72^a^/14.23^b^
Optimum moisture content, OMC (%)	39^a^/27^b^
Unified soil classification system	High plastic clay
Main minerals (%)	M = 61
	Q = 25
	C = 10
	B = 2

*M* montmorillonite, *Q* quartz, *B* biotite, *C* calcite.

^a^Standard A compaction test, ^b^modified compaction test, *methylene blue adsorption^24^.

For the preparation of soil samples, oven-dried bentonite was poured into a consolidation mold and directly compacted to achieve initial dry unit weights of 11.77, 12.75, and 13.73 kN/m^3^ with an initial moisture content of 5%. Note these three different target unit weights were determined based on both standard and modified compaction tests. After completing the compaction of the soil sample, the soil sample was moved into a base container and saturated with NaCl solutions with molar concentrations of 0 (i.e. DI water), 0.1, 0.5, and 1.0 M.

### Test procedures during swelling

**Figure 1:**
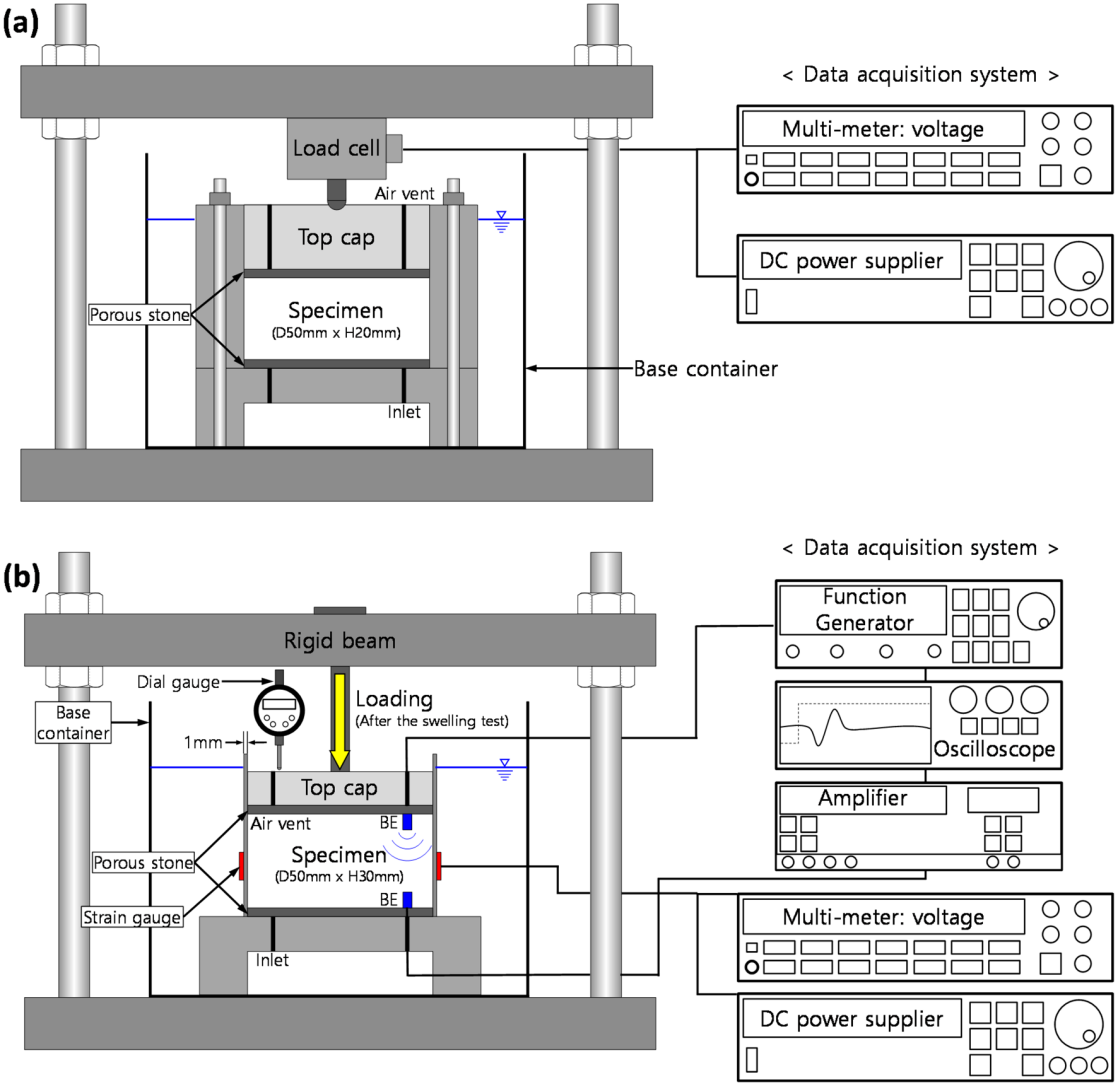
Experimental setup of: (**a**) vertical swelling pressure test and (**b**) horizontal swelling pressure test (loading test after swelling). *BE*  bender elements.

During the expansion of compacted bentonite, vertical and horizontal swelling pressure tests were performed separately. Figure 1a shows the experimental setup of the vertical swelling pressure test. After the completion of the sample preparation for the vertical swelling pressure test (sample dimension: diameter of 50 mm and height of 20 mm), the vertical swelling pressure generated in the process of pore water infiltration into the sample was directly measured by a load cell.

The horizontal swelling pressure tests were conducted with a customized thin-wall oedometer cell instead of a conventional oedometer cell (Fig. 1b). The thin-wall oedometer cell was made of stainless steel, which has an inner diameter of 50 mm and a wall thickness of 1 mm. The maximum horizontal stress that can be applied to the employed thin-wall oedometer cell is 400 kPa to ensure the *K*_*0*_ condition (i.e. lateral strain < 5∙10^–5^^27^. Two strain gauges were symmetrically installed on the outside of the thin-wall oedometer cell at the middle-height of the soil sample (15 mm) to measure the horizontal swelling pressure, and two dummy gauges were also employed for temperature compensation. Before performing the horizontal swelling pressure test, the strain gauges were calibrated using a balloon filled with water to obtain the correlation between the horizontal stress and output voltage (see details in^19^. The soil sample for the horizontal swelling pressure test was prepared using the same procedures as those for the vertical swelling pressure test (sample dimension: diameter of 50 mm and height of 30 mm). During both the vertical and horizontal swelling pressure tests, the volume of the sample was constantly maintained.

For the measurement of shear wave velocity, the bender elements (source and receiver elements) were installed at the bottom of the thin-wall oedometer cell and at the top cap (Fig. 1b). The square wave with frequency = 20 HZ and amplitude = 10 V, generated by a function generator (Agilent, 34970A), propagated through the specimen. The received signal was filtered and amplified by a filter amplifier (Krohn-Hite, 3364), and the filtered and amplified signal was digitized by an oscilloscope (Agilent, 54624A). To eliminate random noise, 1024 received signals were averaged. The tip-to-tip distance between bender elements was used as the travel distance, and the first arrival time was chosen from the recorded data in consideration of the near-field effect (see details in^28^. The shear wave velocity was continuously measured until the swelling was completed.

### Test procedures after swelling

After completing the horizontal swelling test with the thin-wall oedometer cell, the soil sample was loaded to investigate the characteristics of compacted bentonite after swelling. The vertical stress was gradually loaded up to 297 kPa and the horizontal stress was measured by using strain gauges, as mentioned earlier. Each loading step lasted for 24 h and the shear wave velocity was obtained at the end of each loading step. In addition, the deformation of the soil sample during the loading tests was measured using a digital dial gauge attached to the top cap, and subsequently, the void ratio was calculated to investigate the deformation characteristics of compacted bentonite after swelling.

## Results and discussion

### Measurements during swelling

#### Vertical and horizontal swelling pressures

**Figure 2:**
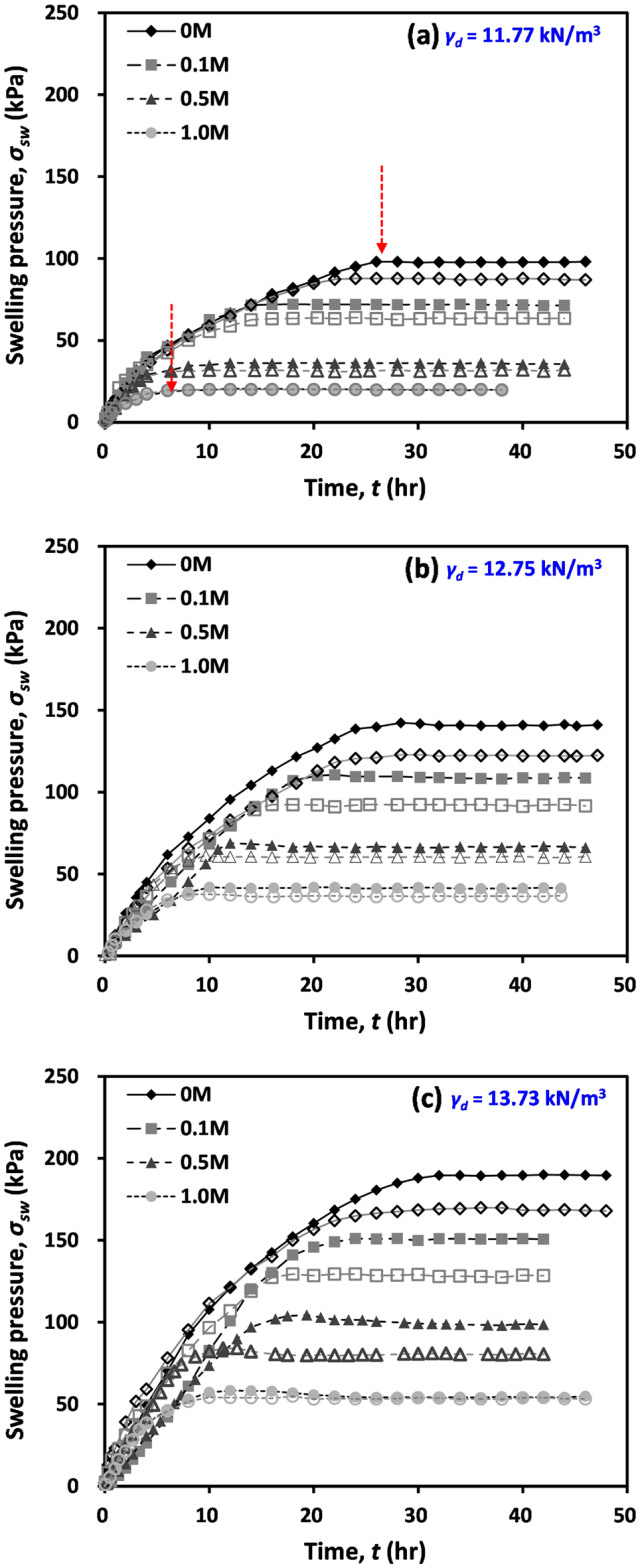
Evolution of vertical and horizontal swelling pressures with respect to time for varying NaCl concentrations: (**a**) specimens with initial dry unit weight *γ*_*d*_ = 11.77 kN/m^3^; (**b**) *γ*_*d*_ = 12.75 kN/m^3^; and (**c**) *γ*_*d*_ = 13.73 kN/m^3^. In the figure, the vertical swelling pressures are presented by filled symbols, and hollow symbols indicate the horizontal swelling pressures.

Figure 2a–c show the variations in the vertical and horizontal swelling pressures (*σ’*_*sw*_) of compacted bentonite with increasing time during swelling at a constant volume with varying NaCl concentrations for initial dry unit weights of 11.77, 12.75, and 13.73 kN/m^3^, respectively. During the swelling process, a significant increase in *σ’*_*sw*_ was observed with increasing time at the earlier stage of tests. Then, the rate of change in *σ’*_*sw*_ gradually decreased, and ultimately the *σ’*_*sw*_ reached a constant value.

As the initial dry unit weight of the soil sample increased, the measured *σ’*_*sw*_ increased for all NaCl concentrations (Fig. 2). An increase in the dry unit weight indicates an increase in the quantity of montmorillonite (i.e. swelling clay mineral) at a given volume and the consequent decrease in the distance between swelling particles; therefore, the measured *σ’*_*sw*_ increased with increasing dry unit weight^10,15,16,29–33^. As the NaCl concentration increased, the *σ’*_*sw*_ decreased (Fig. 2). An increase in NaCl concentration results in a decrease in the double layer thickness, which in turn causes *σ’*_*sw*_ to decrease^7–9,17,30,34,35^.

Figure 2 also demonstrates that the measured swelling pressure of tested compacted bentonite with higher pore fluid concentrations approached an asymptotic point at an earlier time during the test than that with lower concentrations (see the arrows in Fig. 2). Because the double layer thickness reduces with increasing NaCl concentrations, it can be postulated that the tested compacted bentonite with a high pore fluid concentration has a shorter water flow path between the inside and outside of the diffuse double layer. Additionally, the hydraulic conductivity increases with increasing pore fluid concentrations^36^. Thus, the swelling process can be ceased at a relatively earlier time for tested bentonite with high pore fluid concentrations.

The vertical and horizontal swelling pressures (*σ’*_*sw*_) of tested bentonites were determined as the final value of the swelling process shown in Fig. 2. The determined vertical and horizontal *σ’*_*sw*_ are summarized in Table 3. Overall, the vertical *σ’*_*sw*_ was higher than the horizontal *σ’*_*sw*_ measured at a constant volume in this study. The determined ratio between the horizontal *σ’*_*sw*_ and vertical *σ’*_*sw*_ ranges from 0.836 to 0.977 (average ratio = 0.890 with a coefficient of variation = 4.62%). This anisotropic swelling pressure can be attributed to the anisotropic microstructure of compacted bentonite, and the determined swelling pressure ratio is comparable with the reported values of previous studies^37^.

**Table 3:** Summary of the final vertical and horizontal swelling pressures.

Dry unit weight (kN/m^3^)	Initial moisture content (%)	NaCl concentration (M)	Swelling pressure (kPa)	
			Vertical swelling pressure	Horizontal swelling pressure
11.77	5	0.0	98.13	87.87
		0.1	72.11	63.99
		0.5	36.4	31.99
		1.0	20.58	20.1
12.75	5	0.0	142.34	122.82
		0.1	110.53	92.48
		0.5	68.86	61.24
		1.0	41.98	38.7
13.73	5	0.0	189.91	169.82
		0.1	151.03	129.39
		0.5	100.53	84.21
		1.0	58.3	54.79

#### Shear wave velocity

**Figure 3:**
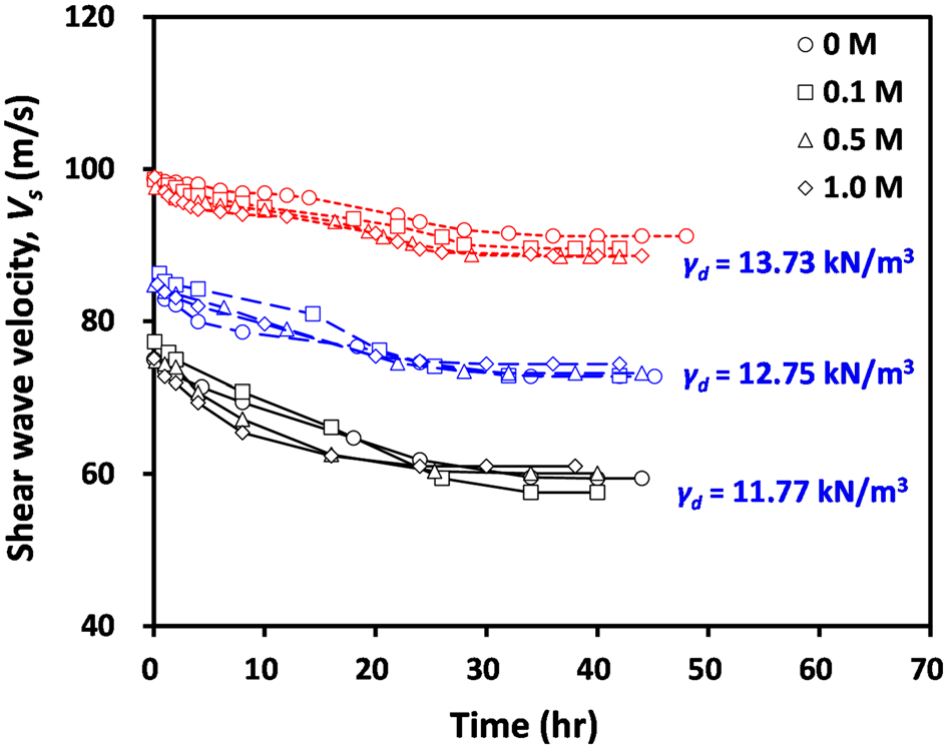
Variation of shear wave velocity of tested compacted bentonites with varying initial dry unit weights and pore fluid concentrations during swelling as a function of time.

Figure 3 shows the measured shear wave velocity (*V*_*s*_) of the tested compacted bentonites with varying pore fluid concentrations and dry unit weights during swelling. Notably, the shear wave velocity of compacted bentonite was measured using the bender element test during the measurement of horizontal swelling pressures under a constant volume (Fig. 1b). As shown in Fig. 3, the measured *V*_*s*_ gradually decreased with time and reached a constant value. With an increase in initial dry unit weight, the measured *V*_*s*_ increased at a given time; however, the effect of pore fluid concentration on the variation of *V*_*s*_ was insignificant (Fig. 3).

The *V*_*s*_ of soils is determined by interparticle contact stiffness and interparticle coordination^24,38–40^. Because an increase in applied stress results in an increase in the interparticle contact area and a consequent increase in contact stiffness between particles, *V*_*s*_ of dry or saturated soils can be expressed as the power function of applied vertical stress (*σ’*_*v*_):

1 $${V}_{s}=\alpha \cdot {\left(\frac{{\sigma }{^\prime}_{v}}{1 kPa}\right)}^{\beta },$$

where *α* and *β* are the fitting parameters. *α* reflects the material constant, strongly influenced by interparticle coordination (or packing state), and *β* indicates the sensitivity of tested materials on applied stress. In the case of unsaturated soils, the matric suction (*u*_*a*_–*u*_*w*_, where *u*_*a*_ = pore air pressure and *u*_*w*_ = pore water pressure) gives an additional increase in interparticle contact stiffness^41^. Thus, Eq. (1) can be modified as^42^:

2 $${V}_{s}=\alpha \cdot {\left(\frac{{\sigma }{^\prime}_{v}}{1 kPa}\right)}^{\beta }+\lambda \cdot \left({u}_{a}-{u}_{w}\right),$$

where *λ* is the fitting parameter, reflecting the packing state and soil type. The vertical stress was not applied during the swelling process in this study; thus, *σ’*_*v*_ in Eq. (2) can be assumed to be 1 kPa (i.e. top cap weight). Consequently, the *V*_*s*_ of tested compacted bentonite during swelling becomes:

3 $${V}_{s}\approx \alpha +\lambda \cdot \left({u}_{a}-{u}_{w}\right).$$

Previous studies^42–44^ clearly demonstrated that the magnitude of matric suction decreases with an increase in the degree of saturation, though this study did not measure the matric suction of tested materials. Therefore, the matric suction in Eqs. (2) or (3) decreases according to swelling process. Furthermore, the generation of swelling pressure results in an increase in the spacing between clay particles^45^. This indicates a decrease in interparticle coordination during swelling and a consequent decrease in *α* in Eqs. (2) or (3). Thus, the measured *V*_*s*_ during swelling decreased with time (Fig. 3).

It is known that a decrease in void ratio (or increase in mass density) results in an increase in matric suction^42^. In addition, the initial packing state of tested soils directly determines the magnitude of *α* in Eq. (3). Therefore, the measured *V*_*s*_ at a given time increases with an increasing initial dry unit weight of tested soils (Fig. 3).

**Figure 4:**
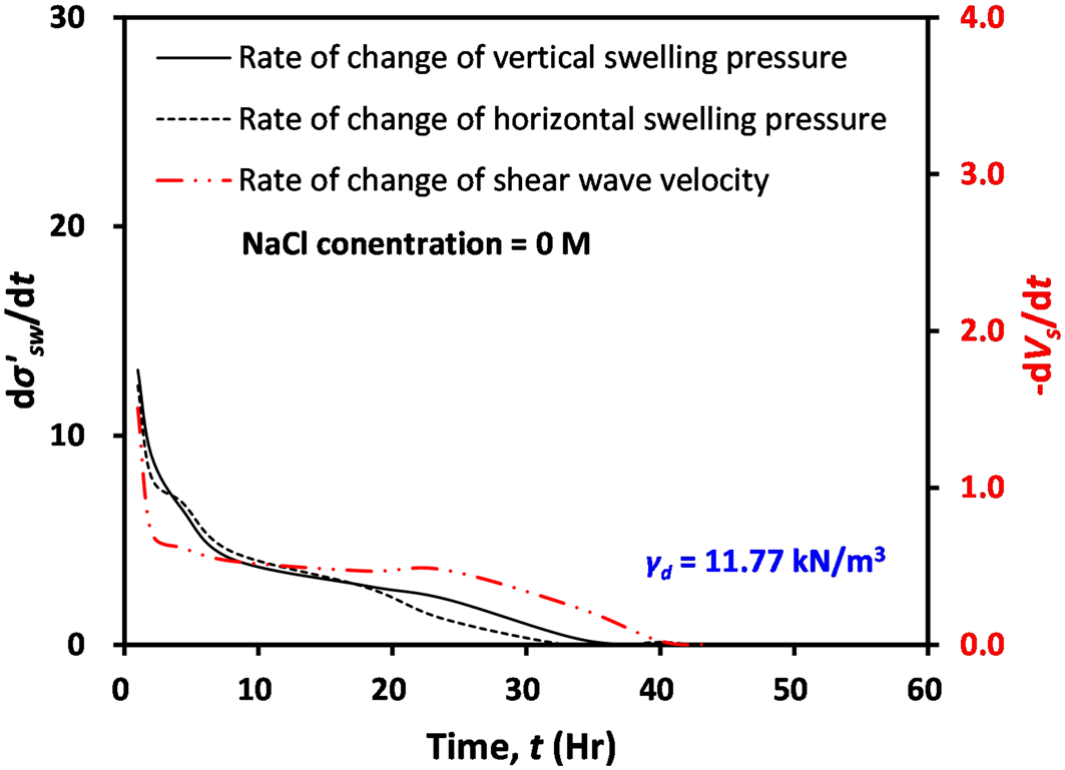
The rate of change in swelling pressure and that in shear wave velocity of tested materials with initial dry unit weight = 11.77 kN/m^3^ and NaCl concentration = 0 M according to time.

Time-dependent variations in both swelling pressure and shear wave velocity appear to be closely related (Figs. 2 and 3). Thus, the rate of changes in the swelling pressures (*dσ’*_*sw*_/*dt*, where *σ’*_*sw*_ = swelling pressure and *t* = time) and shear wave velocity (*dV*_*s*_/*dt*) at a dry unit weight = 11.77 kN/m^3^ and NaCl concentration = 0 M was plotted as a function of time, as shown in Fig. 4. It can be observed in Fig. 4 that the rate of change in *V*_*s*_ resembles that in *σ’*_*sw*_, reflecting that the swelling process or the generation of *σ’*_*sw*_ causes a decrease in *V*_*s*_. In addition, it can be inferred from similar time-dependent behaviors between swelling pressure and shear wave velocity that the measurement of shear wave velocity can be used to determine the initiation and termination of a swelling process.

### Measurements during loading (after swelling)

#### Deformation of compacted bentonite

**Figure 5:**
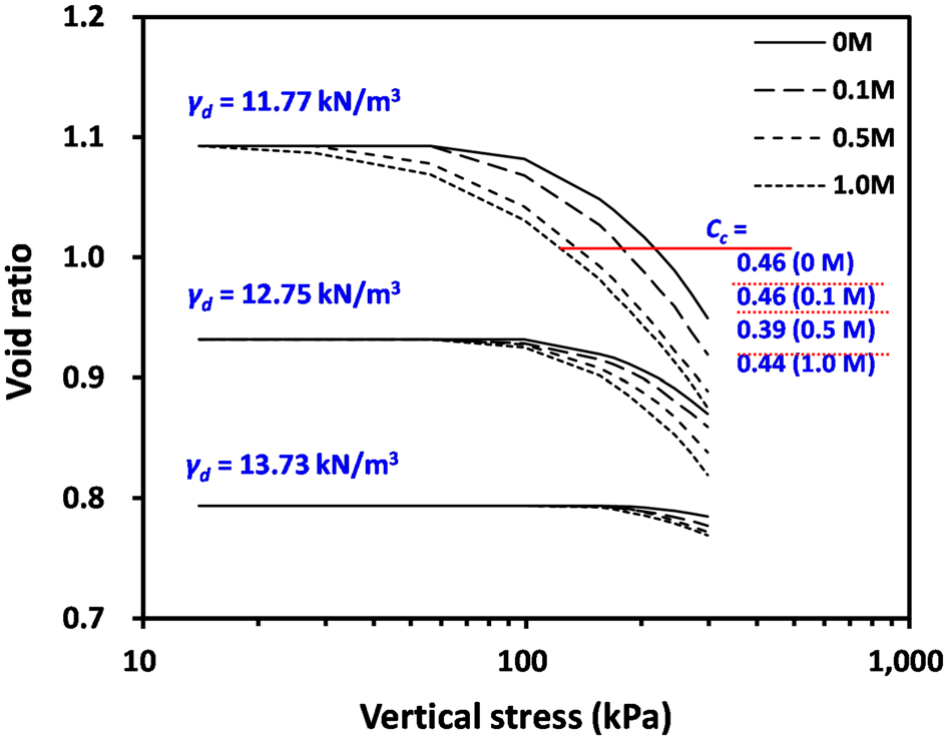
Variation of void ratios of compacted bentonite samples after swelling according to applied vertical stress. *C*_*c*_ compression index.

**Figure 6:**
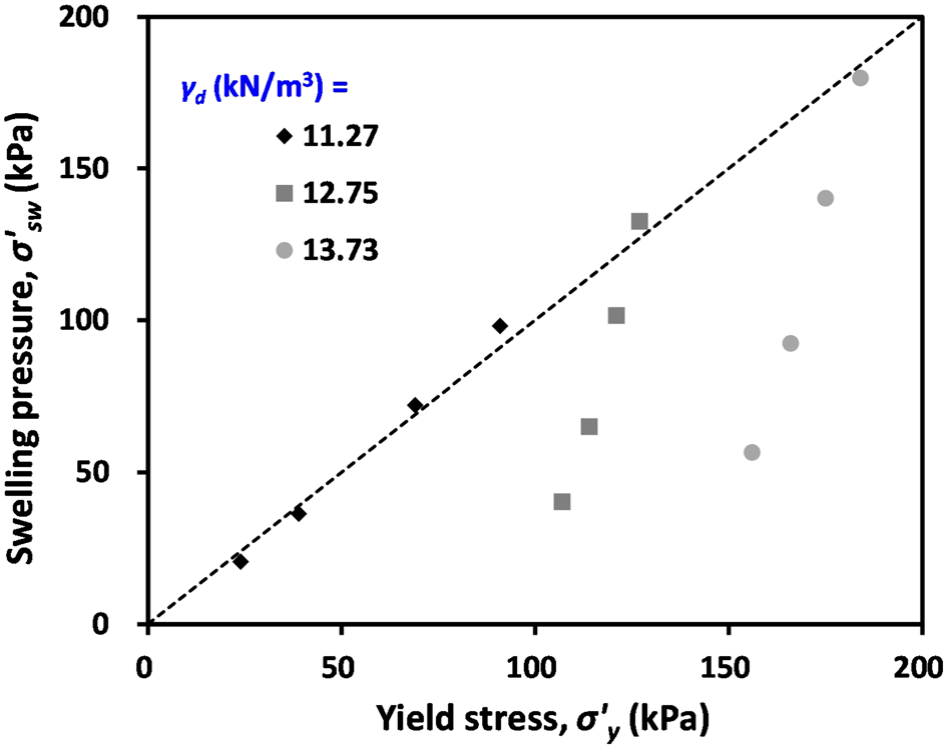
Comparison between swelling pressure and yield stress.

To determine the deformation characteristics of compacted bentonite after the swelling process, the change in void ratio of soil samples was determined as a function of the applied vertical stress, as shown in Fig. 5. Figure 5 clearly demonstrates that the measured void ratio of all tested materials was constant up to certain points of stress levels despite the increasing applied vertical stress. As the applied vertical stress further increased, the void ratio of tested materials started to decrease. The determined applied vertical stress when the void ratio started to decrease (i.e. yield stress *σ’*_*y*_) of tested materials is compared with swelling pressure in Fig. 6. Note that the swelling pressure (*σ’*_*sw*_) in Fig. 6 is the average of vertical and horizontal swelling pressures reported in Table 3. Most notably, Fig. 6 indicates that the *σ’*_*y*_ of the tested sample with initial unit weight (*γ*_*d*_) = 11.77 kN/m^3^ coincides with the *σ’*_*sw*_ in Table 3. By contrast, the *σ’*_*y*_ of tested samples with initial *γ*_*d*_ = 12.75 and 13.73 kN/m^3^ deviates from the *σ’*_*sw*_.

Because of the sample preparation method employed in this study, the tested compacted bentonite experiences compaction-induced yield stress (or preconsolidation stress). In the case of tested specimens with initial *γ*_*d*_ = 11.77 kN/m^3^, the target density can be easily achieved by applying relatively low compaction energy. Thus, in the case of tested materials with *γ*_*d*_ = 11.77 kN/m^3^, the determined *σ’*_*sw*_ was greater than that of compaction-induced *σ’*_*y*_. Consequently, the *σ’*_*sw*_ may act as a preconsolidation stress for the sample with initial *γ*_*d*_ = 11.77 kN/m^3^, causing the compacted bentonite after swelling to show negligible settlement until the applied vertical effective stress is greater than *σ’*_*sw*_. By contrast, the tested specimens with a higher target density required higher compaction energy. Thus, it can be postulated that the compaction-induced *σ’*_*y*_ was greater than *σ’*_*sw*_ in the case of tested materials with initial *γ*_*d*_ = 12.75 and 13.73 kN/m^3^. Relatively narrow variations of *σ’*_*y*_ with varying NaCl concentrations and *σ’*_*y*_ always being greater than *σ’*_*sw*_ support this explanation (Figs. 5 and 6).

The mechanical properties of tested materials with initial *γ*_*d*_ = 12.75 and 13.73 kN/m^3^ involved the impact of compaction-induced yield stress. Thus, to emphasize the sole impact of swelling pressure on the engineering properties of compacted bentonite after swelling, the following sections report the changes in compressibility, lateral stress, and shear wave velocity of compacted bentonite with initial *γ*_*d*_ = 11.77 kN/m^3^ after swelling.

The deformation of soils is the result of a change in effective stress. Thus, it can be assumed that the clay particles in the compacted bentonite after swelling do not experience any increase in effective stress until the initiation of deformation because the internal swelling pressure cancels out the external stress. Consequently, the external stress (or applied vertical effective stress, *σ’*_*v*_) exceeding the swelling pressure (*σ’*_*sw*_) will cause an increase in effective stress of clay particles, leading to the deformation (void ratio change, Δ*e*) of compacted bentonite after swelling in Fig. 5 can be expressed as:

4 $$\Delta e={C}_{c}\cdot log\left(\frac{{\sigma }{^\prime}_{v}}{{\sigma }{^\prime}_{sw}}\right),\mathrm{ when\,}\sigma {^\prime}_{v} > \sigma {^\prime}_{sw}$$

where *C*_*c*_ denotes the compression index. Note, Eq. (4) is identical to the inverse of the equation for calculating heave from the constant swell test^46^. The determined *C*_*c*_ values are given in Fig. 5, and the *C*_*c*_ values for tested materials with initial *γ*_*d*_ = 11.77 kN/m^3^ are comparable to those in the authors’ previous study on saturated bentonite with similar void ratio^47^, reflecting that the deformation characteristics of compacted bentonite after swelling may be similar to those of saturated bentonite without swelling.

Figure 5 also demonstrates the effect of NaCl concentrations on the compressibility of tested compacted bentonite after swelling: the void ratio at a given applied stress level decreases with increasing NaCl concentration. Because swelling pressure (or *σ’*_*y*_) decreases with increasing NaCl concentrations (Figs. 2 and 5), the specimen with higher pore fluid concentration can start to deform at lower stress levels, leading to a greater change in the void ratio according to Eq. (4). By contrast, the impact of pore fluid concentration on *C*_*c*_ values was insignificant (Fig. 5).

#### Applied vertical stress and measured horizontal stress

**Figure 7:**
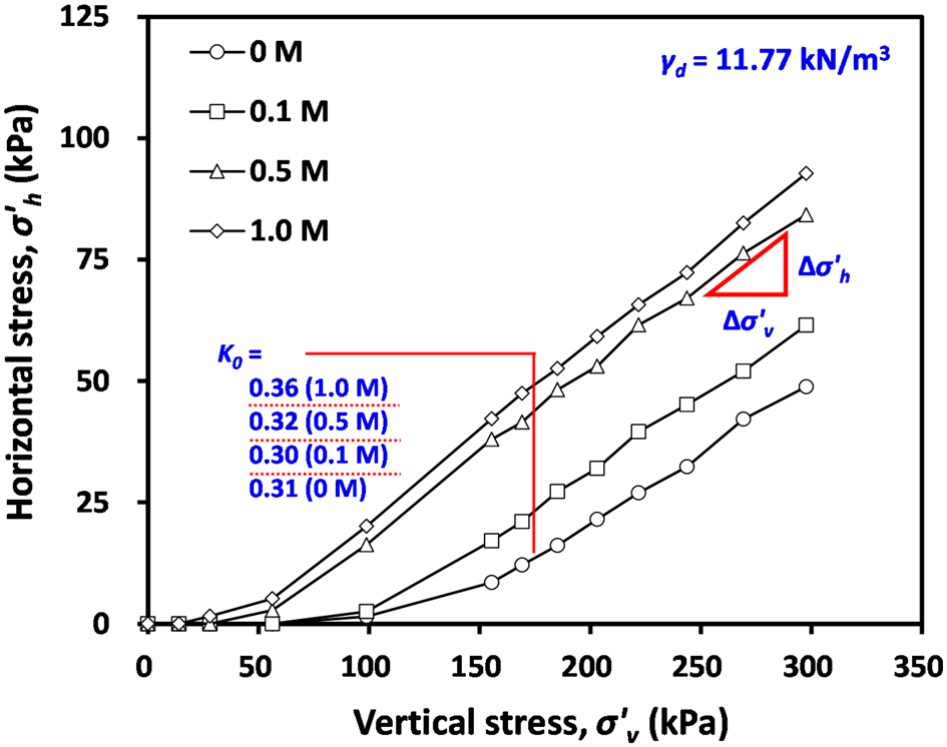
Variation in the measured horizontal stress with increasing applied vertical stress for tested compacted bentonite with initial *γ*_*d*_ = 11.77 kN/m^3^ after swelling.

Figure 7 presents the variation in the measured horizontal stress with increasing applied vertical stress for tested compacted bentonite with initial *γ*_*d*_ = 11.77 kN/m^3^ after swelling. It can be observed in Fig. 7 that, despite the increase in vertical stress on the sample, the horizontal stress remained unchanged up to certain points of stress levels. After passing certain stress levels, the horizontal stress started to increase, and Fig. 7 clearly indicates that the stress level for the initiation of lateral stress increases coincides with the swelling pressure. This observation reconfirms that the swelling pressure cancels out the externally applied stress; thus, the change in lateral stress was only measurable when the applied vertical stress was greater than swelling pressure.

From the change in measured horizontal stress (Δ*σ’*_*h*_) and that in applied vertical stress (Δ*σ’*_*v*_), the coefficient of lateral earth stress at-rest (*K*_*0*_) was calculated according to (triangle in Fig. 7):

5 $${K}_{0}=\frac{{\Delta \sigma }{^\prime}_{h}}{{\Delta \sigma }{^\prime}_{v}}, \mathrm{when\,}\sigma {^\prime}_{v} > \sigma {^\prime}_{sw}$$

The determined *K*_*0*_ is given in Fig. 7, and it can be observed in Fig. 7 that the effect of pore fluid concentration on the determined *K*_*0*_ was insignificant. Thus, high lateral stress at a given applied vertical stress with increasing NaCl concentration can be attributed to the small swelling pressure with increasing pore fluid concentration.

*K*_*0*_ of saturated clay generally ranges from 0.5 to 1, which is greater than the determined *K*_*0*_ of tested materials after swelling. It is known that the wetting of compacted bentonite with inundation may not ensure the fully saturated condition of compacted bentonite^48^,thus, the tested compacted bentonite after swelling may contain some portion of matric suction. Note that the *K*_*0*_ of unsaturated clay based on elasticity theory can be expressed as a function of matric suction (*u*_*a*_–*u*_*w*_)^49^ as follows:

6 $${K}_{0}=\frac{\mu }{1-\mu }-\frac{1-2\mu }{1-\mu }\cdot \frac{\mathrm{\rm X}\cdot \left({u}_{a}-{u}_{w}\right)}{\left({\sigma }_{v}-{u}_{a}\right)},$$

where *Χ* = fitting parameter, ranging from 0 to 1. Thus, with an increase in matric suction (or with a decrease in the degree of saturation), the *K*_*0*_ decreases according to Eq. (6), leading to the measured *K*_*0*_ of tested compacted bentonite after swelling (Fig. 7) being smaller than the typical *K*_*0*_ of saturated clay.

#### Shear wave velocity (V_s_)

**Figure 8:**
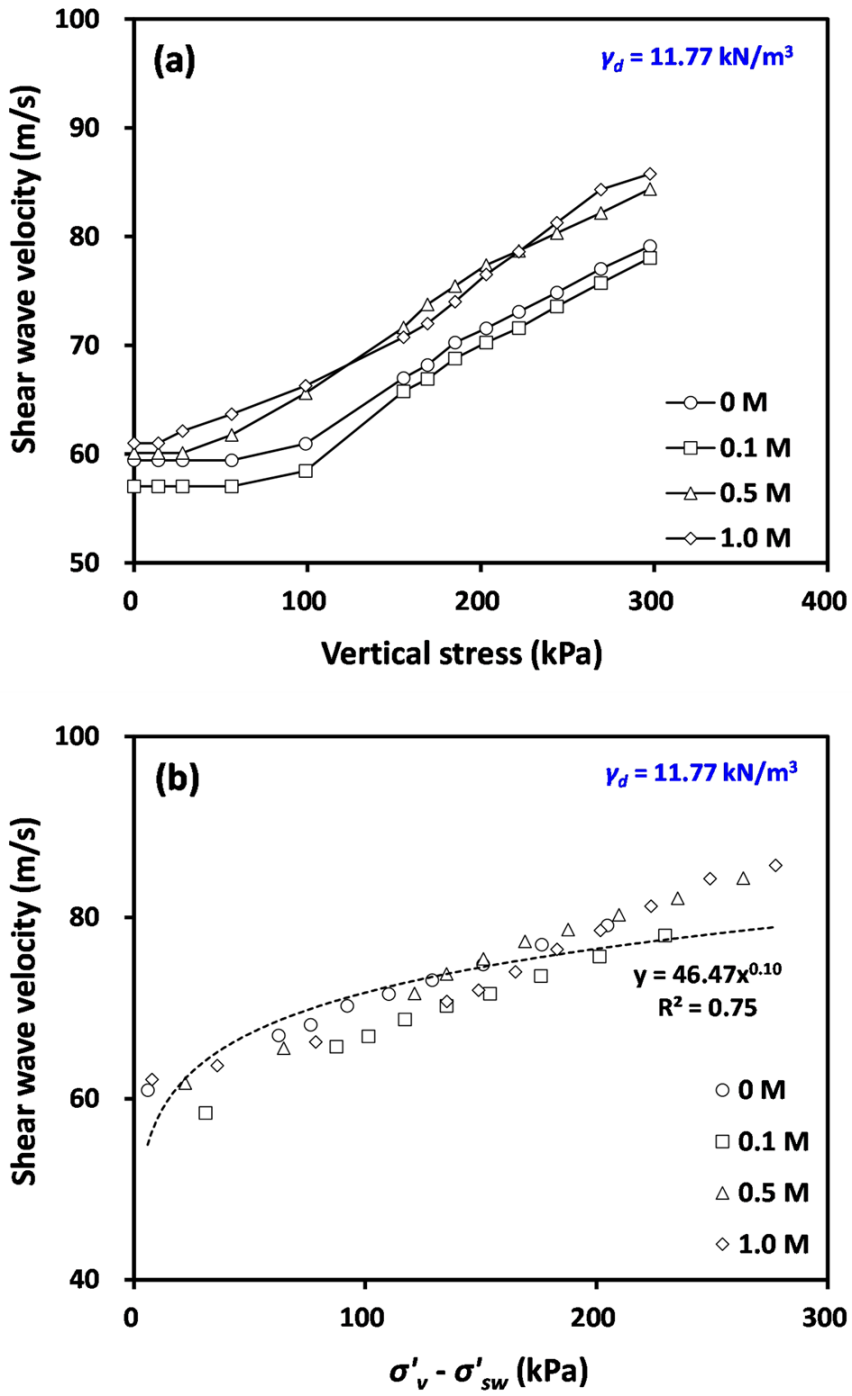
Variations of shear wave velocity of tested compacted bentonite after swelling according to (**a**) applied vertical stress and (**b**) difference between applied vertical stress and swelling pressure.

**Figure 9:**
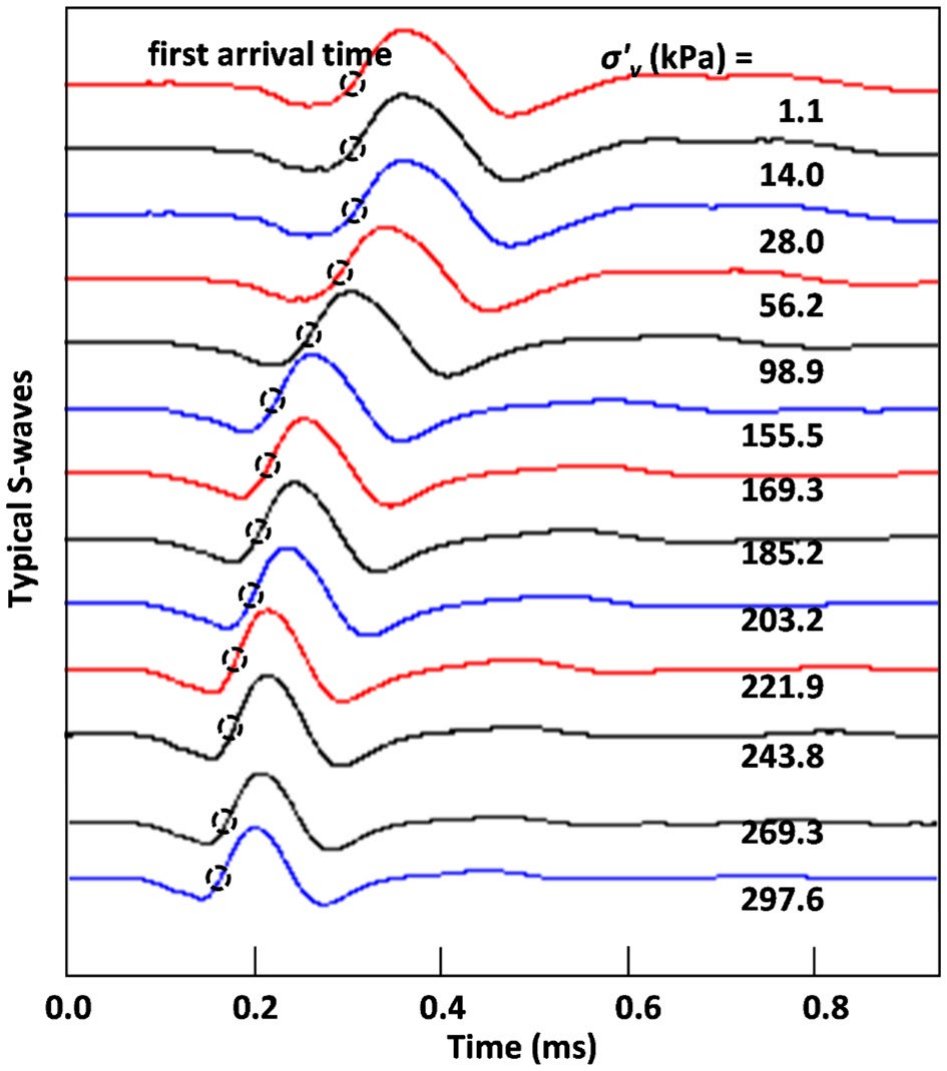
Measured shear waves with loading for compacted bentonite with initial dry unit weight = 11.77 kN/m^3^ and NaCl concentration = 0 M after swelling.

Figure 8a shows the measured shear wave velocity (*V*_*s*_) of compacted bentonite with initial *γ*_*d*_ = 11.77 kN/m^3^ according to applied vertical stress. Exhibiting similarity to the results of deformation (Fig. 5) and lateral stress (Fig. 7), *V*_*s*_ did not increase until the applied vertical effective stress (*σ’*_*v*_) approached the swelling pressure (*σ’*_*sw*_), reflecting a negligible change in interparticle contact stiffness when *σ’*_*v*_ was smaller than *σ’*_*sw*_. Figure 9 shows the measured shear waves with loading steps for compacted bentonite with initial dry unit weight (*γ*_*d*_) = 11.77 kN/m^3^ and NaCl concentration = 0 M. Figure 9 also clearly indicates that the change in first arrival time (dotted circle in Fig. 9) was only measurable when the *σ’*_*v*_ approached *σ’*_*sw*_. When *σ’*_*v*_ exceeded *σ’*_*sw*_, *V*_*s*_ increased with increasing applied vertical stress. Thus, the *V*_*s*_ of tested materials after swelling can be expressed as:

7 $${V}_{s}={\alpha }{^\prime}\cdot {\left(\frac{{\sigma }{^\prime}_{v}-{\sigma }{^\prime}_{sw}}{1 kPa}\right)}^{\beta }.$$

Figure 8b shows the variation of *V*_*s*_ as a function of (*σ’*_*v*_–*σ’*_*sw*_) (Eq. (7)). Tested compacted bentonite after swelling shows very comparable *V*_*s*_ at a given (*σ’*_*v*_–*σ’*_*sw*_) regardless of pore fluid concentrations, reflecting that a higher *V*_*s*_ at a given applied stress with increasing NaCl concentration in Fig. 8a can be attributed to the earlier initiation of *V*_*s*_ increase with increasing pore fluid concentration resulting from a smaller swelling pressure. The typical stress exponent *β* of dry or saturated soils is approximately 0.25. However, the determined *β* of tested compacted bentonite after swelling is approximately 0.1, implying that the measured *V*_*s*_ after swelling still includes the contribution from the matric suction (Eq. (2)). Thus, this smaller* β* of tested compacted bentonite after swelling compared to typical dry or saturated soils reinforces that the tested material is not fully saturated after swelling.

In summary, in the case where the applied vertical stress (*σ’*_*v*_) is smaller than swelling pressure (*σ’*_*sw*_), the change in the internal effective stress of soils is minimal (Fig. 7). Therefore, the tested compacted bentonite after swelling shows a negligible change in compressibility and shear wave velocity when *σ’*_*v*_ is smaller than *σ’*_*sw*_. When there is a change in internal effective stress (i.e. *σ’*_*v*_ > *σ’*_*sw*_), the tested soils show a change in mechanical properties. This in turn demonstrates that the continuous measurement of shear wave velocity can provide insight into the change in internal effective stress of compacted bentonite after swelling. Thus, the change in engineering properties of compacted bentonite after swelling can be determined through the measurement of shear wave velocity.

## Conclusion

This study experimentally investigated the characteristics of compacted bentonite during and after swelling using the measurement of shear wave velocity (*V*_*s*_). The compacted bentonite specimens with varying dry unit weights were prepared under varying NaCl pore fluid concentrations. The vertical and horizontal swelling pressures and *V*_*s*_ of tested compacted bentonite were measured during the swelling process at a constant volume. In addition, the soil sample was loaded to investigate the variations of the deformation, lateral stress, and *V*_*s*_ of the compacted bentonite after swelling. The key findings of this study are as follows:

1. The swelling pressure (*σ’*_*sw*_) increased with increasing dry unit weight of tested materials because of an increase in the quantity of swelling clay mineral at a given volume. In addition, the *σ’*_*sw*_ increased with decreasing pore fluid concentrations because of an increase in double layer thickness.
2. Time-dependent variation of *V*_*s*_ resembles that of *σ’*_*sw*_, reflecting that the measurement of shear wave velocity can be used to determine the initiation and termination of a swelling process.
3. The changes in lateral stress and void ratio of compacted bentonite after swelling were only measurable when the applied vertical stress was greater than the swelling pressure, reflecting that the swelling pressure cancels out the externally applied stress.
4. Because *V*_*s*_ is mainly determined by interparticle stiffness, which is the function of the internal effective stress of soils, the measurement of *V*_*s*_ can capture the change in engineering properties of compacted bentonite after swelling.

## Data Availability

All data generated or analyzed during this study are available from the corresponding author on reasonable request.
